# Supplementary material for: Origin of Bluetongue Virus Serotype 8 Outbreak in Cyprus, September 2016
Source: Viruses. 2020 Jan 14;12(1):96. doi: 10.3390/v12010096 (PMC7019704; doi:10.3390/v12010096)
Supplement: Supplementary file 1 [file viruses-12-00096-s001.zip › Table S1.pdf]

Table S1. Historical reference strains of BTV sequenced during this study and associated Genbank accession number for the genome segments.

Isolate /Serotype	Passage sequenced	Seg-1 (VP1)	Seg-2 (VP2)	Seg-3 (VP3)	Seg-4 (VP4)	Seg-5 (NS1)	Seg-6 (VP5)	Seg-7 (VP7)	Seg-8 (NS2)	Seg-9 (VP6)	Seg-10 (NS3)
RSArrrr/05	E2BHK7	MN710093	MN710209	MN710122	MN710151	MN710180	MN710238	MN710267	MN710296	MN710325	MN710354
RSArrrr/07	E1BHK10	MN710094	MN710210	MN710123	MN710152	MN710181	MN710239	MN710268	MN710297	MN710326	MN710355
RSArrrr/10	BHK7	MN710095	MN710211	MN710124	MN710153	MN710182	MN710240	MN710269	MN710298	MN710327	MN710356
RSArrrr/11	E1BHK7	MN710096	MN710212	MN710125	MN710154	MN710183	MN710241	MN710270	MN710299	MN710328	MN710357
RSArrrr/12	E1BHK8	MN710097	MN710213	MN710126	MN710155	MN710184	MN710242	MN710271	MN710300	MN710329	MN710358
RSArrrr/13	E1/BHK7	MN710098	MN710214	MN710127	MN710156	MN710185	MN710243	MN710272	MN710301	MN710330	MN710359
RSArrrr/17	S3/E2/LK7/BHK11	MN710099	MN710215	MN710128	MN710157	MN710186	MN710244	MN710273	MN710302	MN710331	MN710360
RSArrrr/18	E1/BHK8	MN710100	MN710216	MN710129	MN710158	MN710187	MN710245	MN710274	MN710303	MN710332	MN710361
RSArrrr/19	E3/BHK8	MN710101	MN710217	MN710130	MN710159	MN710188	MN710246	MN710275	MN710304	MN710333	MN710362
RSArrrr/20	E2/BHK1/CER1/BHK5	MN710102	MN710218	MN710131	MN710160	MN710189	MN710247	MN710276	MN710305	MN710334	MN710363
RSArrrr/21	V3/BHK16	MN710103	MN710219	MN710132	MN710161	MN710190	MN710248	MN710277	MN710306	MN710335	MN710364
RSArrrr/22	E2/BHK1/CER1/BHK5	MN710104	MN710220	MN710133	MN710162	MN710191	MN710249	MN710278	MN710307	MN710336	MN710365
RSArrrr/23	E1/CER2/BHK5	MN710105	MN710221	MN710134	MN710163	MN710192	MN710250	MN710279	MN710308	MN710337	MN710366
