# Supplementary figures and images for: Origin of Bluetongue Virus Serotype 8 Outbreak in Cyprus, September 2016
Source: Viruses. 2020 Jan 14;12(1):96. doi: 10.3390/v12010096 (PMC7019704; doi:10.3390/v12010096)

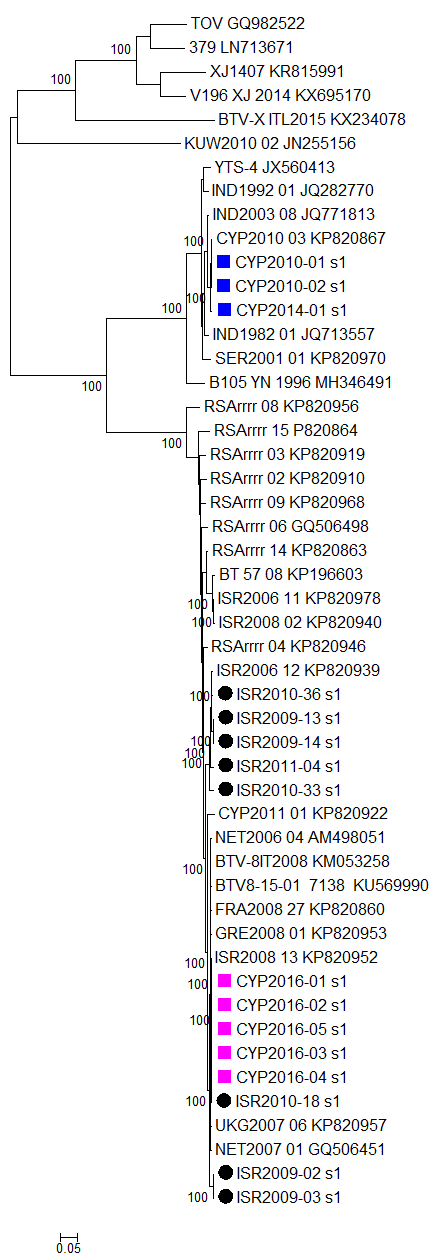

Supplement: Supplementary file 1 [file viruses-12-00096-s001.zip › Figure S1a.png]

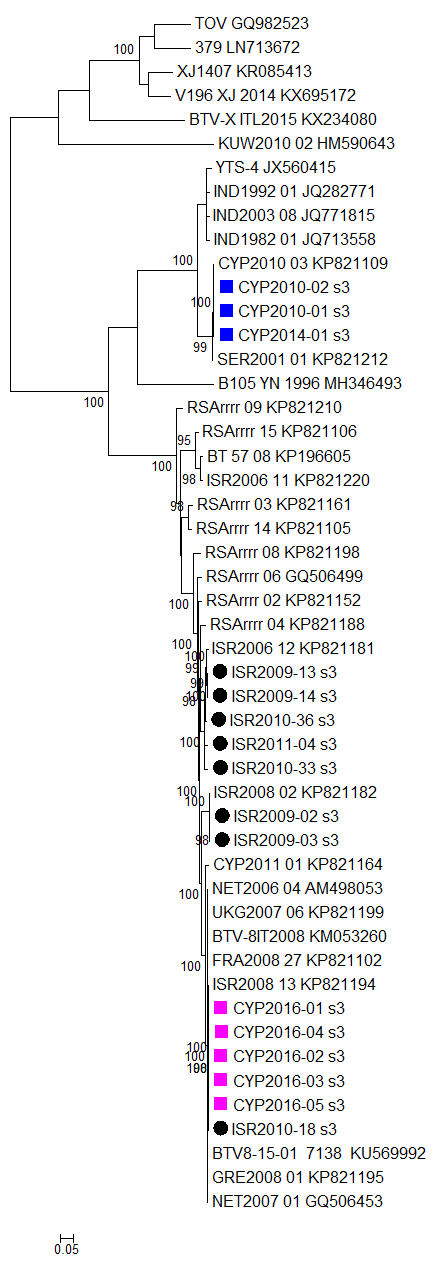

Supplement: Supplementary file 1 [file viruses-12-00096-s001.zip › Figure S1b.png]

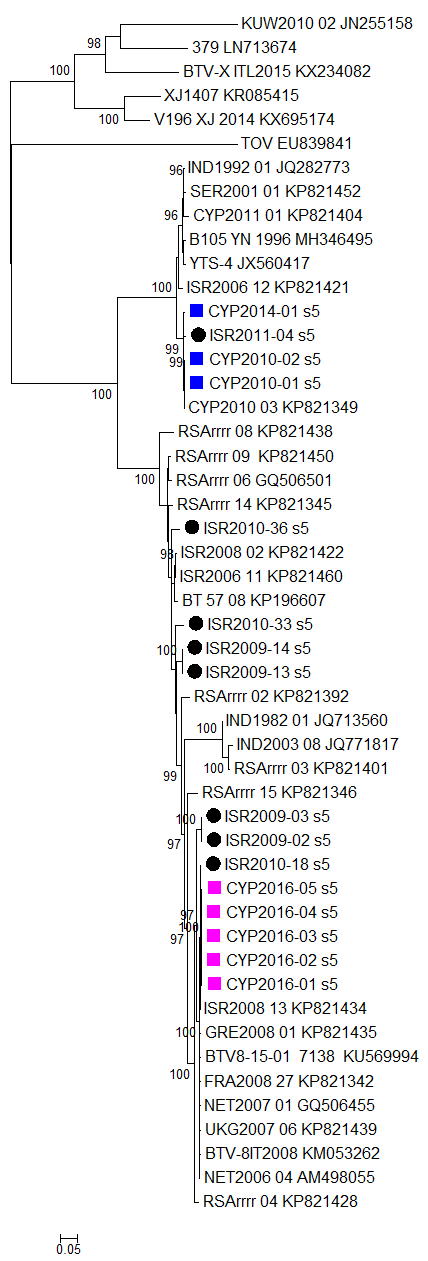

Supplement: Supplementary file 1 [file viruses-12-00096-s001.zip › Figure S1c.png]

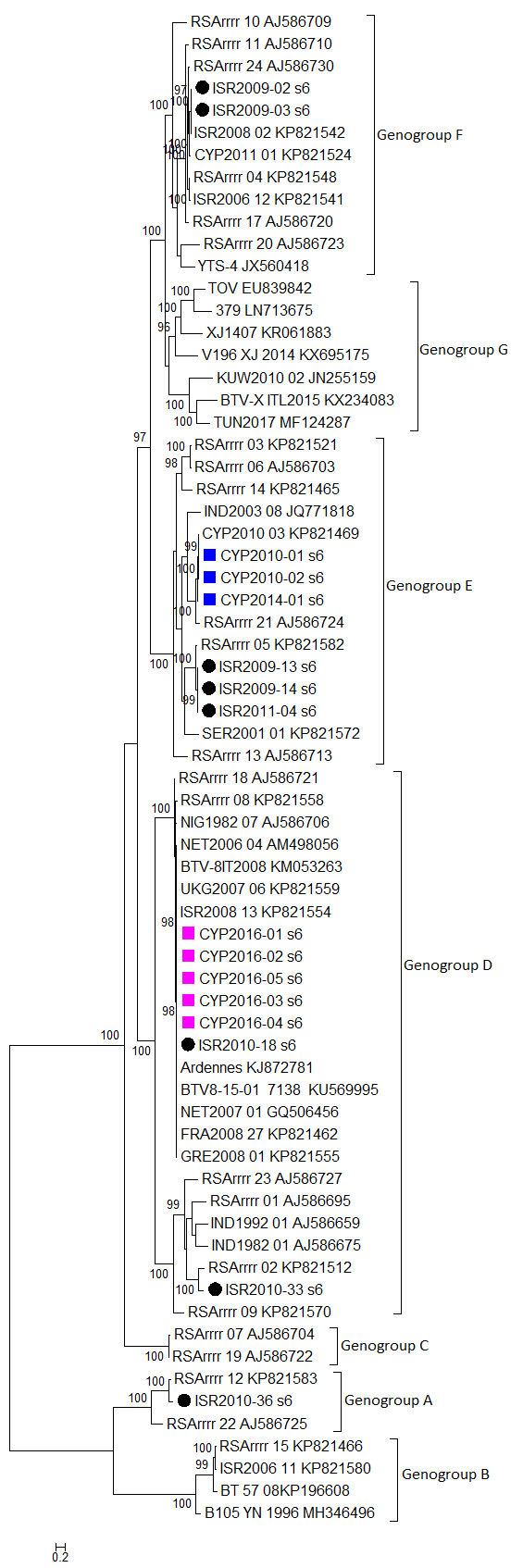

Supplement: Supplementary file 1 [file viruses-12-00096-s001.zip › Figure S1d.png]

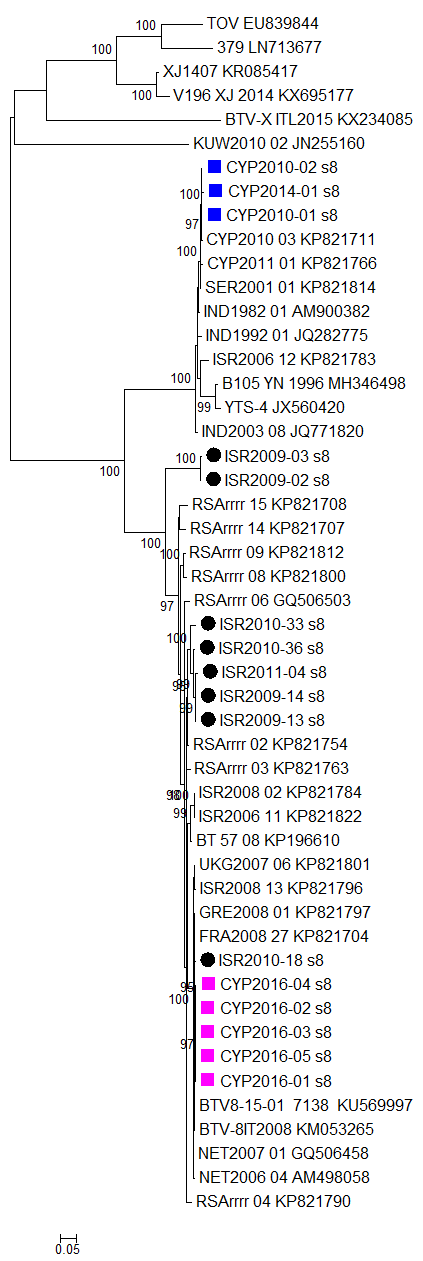

Supplement: Supplementary file 1 [file viruses-12-00096-s001.zip › Figure S1e.png]
